# Targeting functionalized nanoparticles to activated endothelial cells under high wall shear stress

**DOI:** 10.1002/btm2.10151

**Published:** 2019-12-13

**Authors:** Hila Zukerman, Maria Khoury, Yosi Shammay, Josué Sznitman, Noah Lotan, Netanel Korin

**Affiliations:** ^1^ Department of Biomedical Engineering Technion – Israel Institute of Technology Haifa Israel

**Keywords:** drug delivery, endothelium, inflammation, microfluidics, shear stress, vascular‐targeted carriers (VTCs)

## Abstract

Local inflammation of the endothelium is associated with a plethora of cardiovascular diseases. Vascular‐targeted carriers (VTCs) have been advocated to provide focal effective therapeutics to these disease sites. Here, we examine the design of functionalized nanoparticles (NPs) as VTCs that can specifically localize at an inflamed vessel wall under pathological levels of high shear stress, associated for example with clinical (or in vivo) conditions of vascular narrowing and arteriogenesis. To test this, carboxylated fluorescent 200 nm polystyrene particles were functionalized with ligands to activated endothelium, that is, an E‐selectin binding peptide (Esbp), an anti ICAM‐1 antibody, or using a combination of both. The functionalized NPs were investigated in vitro using microfluidic models lined with inflamed (TNF‐α stimulated) and control endothelial cells (EC). Specifically, their adhesion was monitored under different relevant wall shear stresses (i.e., 40–300 dyne/cm^2^) via real‐time confocal microscopy. Experiments reveal a significantly higher specific adhesion of the examined functionalized NPs to activated EC for the window of examined wall shear stresses. Moreover, particle adhesion correlated with the surface coating density whereby under high surface coating (i.e., ~10,000 molecule/particle), shear‐dependent particle adhesion increased significantly. Altogether, our results show that functionalized NPs can be designed to target inflamed endothelial cells under high shear stress. Such VTCs underscore the potential for attractive avenues in targeting drugs to vasoconstriction and arteriogenesis sites.

**Abbreviations**
aICAM‐1: Anti intracellular adhesion molecule 1
EC: endothelial cells
ECM: endothelial cell media
Esbp: E selectin binding peptide
FBS: fetal bovine serum
HUVEC: human umbilical vein endothelial cells
ICAM‐1: intracellular adhesion molecule 1
NPs: nanoparticles
PBS: phosphate buffered saline
PDMS: polydimethylsiloxane
TNF‐α: tumor necrosis factor alpha
VCAM‐1: vascular cell adhesion molecule 1
VTCs: vascular‐targeted carriers
WSS: wall shear stress

## 1 INTRODUCTION

A variety of cardiovascular diseases, including atherosclerosis, thrombosis, and vasospasm, are characterized by vascular wall inflammation,^1^ where the inflammatory processes play a major role in vascular pathogenesis. The inflammatory reaction is a complex process involving the immune system cells (e.g., lymphocytes, monocytes, macrophages) and the endothelial cell layer veering the vascular walls.^1^ The basic interaction between these two cells types at the initial steps of inflammation relies on inflammatory receptor‐ligand interactions,^2^ and therefore, can be employed for drug delivery using vascular‐targeted carriers (VTCs).

**Figure 1:**
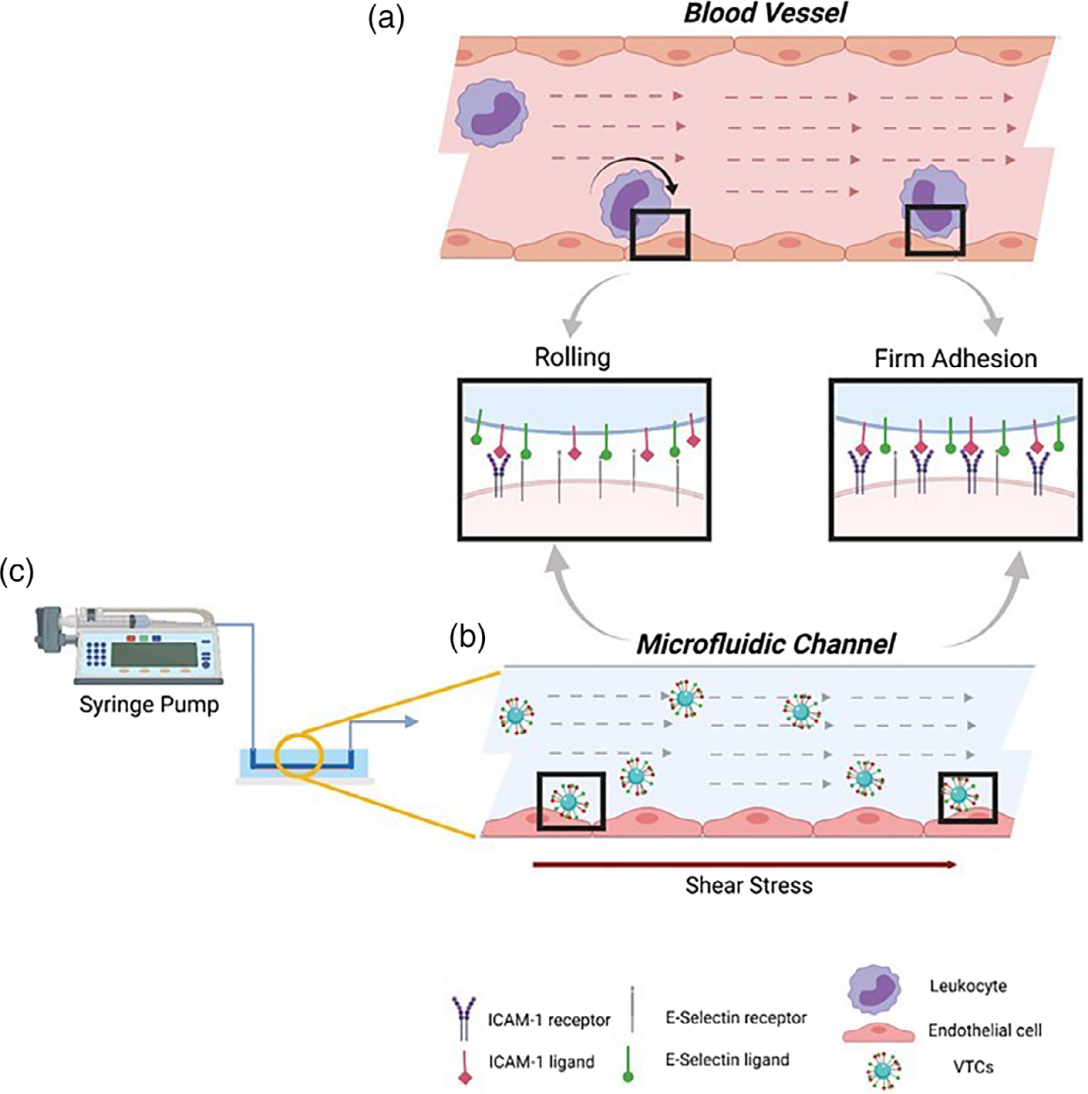
Schematic of adhesion process of (a) leukocyte and (b) leukocyte mimetics VTC to activated EC under flow, as mediated via EC inflammation receptors (including ICAM 1 and E‐selectin) that are locally over‐expressed upon inflammation. (c) The VTCs are perfused by a syringe pump with controlled flow rate inducing shear stress on the seeded EC in a microfluidic channel. The illustration was created with http://biorender.com

Endothelial cells (EC) response to inflammatory mediators is termed endothelial activation and it varies according to the specific mediator. For example, histamine is known to promote short‐term response of ECs, while TNF‐α stimulates a long‐term response.^3^ While short‐term EC response occurs within a few minutes and involves rapid expression of membrane receptors, long‐term response induces new adhesion molecules synthesis.^3^ The inflammatory receptors interact with their ligands on leukocytes surface and can similarly be utilized for targeted drug delivery to activated ECs. The first receptors to be expressed on ECs are receptors of the selectin superfamily. One of them, E‐selectin, is crucial for capturing leukocytes circulating within blood flow and enables their rolling over the inflamed EC under physiological flow^4^, ^5^, ^6^ (see schematic of Figure 1a). E‐selectin is stored in α granules inside resting EC, but following an inflammatory stimuli these vesicles fuse rapidly (within seconds) with the cell membrane.^6^ However, E‐selectin expression declines generally within the first 24 hr post stimulation.^6^ This rapid post‐stimulation expression of E‐selectin may be utilized for efficient drug‐targeting.^7^, ^8^ The next step in EC‐leukocyte cascade, following rolling, consists of a firm adhesion^3^, ^9^, ^10^ as shown in Figure 1b. This step is mitigated by overexpression of immunoglobulins superfamily members, such as Intra‐cellular adhesion molecule‐1 (ICAM‐1) and vascular cell adhesion molecule‐1 (VCAM‐1).^3^, ^11^, ^12^ In contrast to E‐selectin, ICAM‐1 has abroad distribution in the body due to its function in cell–cell and cell‐matrix adhesion. While under resting conditions ICAM‐1 expression is low, upon chemokines simulation or shear stress alterations^6^, ^12^ it is upregulated. ICAM‐1 becomes overexpressed mostly after 4 hr, and even 48 hr later there is still altered expression of the ICAM‐1 receptor.^13^ Yet, the stable pairing of the ICAM‐1 receptor to its ligands provides firm adhesion, which is highly desirable in drug delivery approach.^7^, ^8^

Based on the natural molecular interaction occurring during inflammation between leukocyte and EC, vascular‐targeted carriers (VTCs) that target activated ECs have been sought and developed (Figure 1b) as a potent drug delivery strategy.^14^ Briefly, the active targeting of VTCs is based on functionalizing these carriers with ligands and antibodies that enable specific ligand‐receptor pairing with their complimentary overexpressed inflammatory receptors on the EC.^13^, ^15^ Achieving effective targeting remains a challenging task where VTCs are designed to adhere to specific receptor overexpressed on a limited number of cells at the disease site while avoiding adhesion to vascular cells throughout the body, where cells exhibit lower receptor expression.^16^ Therefore, various studies have focused on targeting functionalized nanoparticles to inflamed endothelium based on over‐expression of adhesion receptors such as: ICAM‐1, VCAM, and E‐selectin.^17^, ^18^ However, in the cardiovascular system, this task needs to be performed under dynamic conditions including blood flow and its associated hemodynamic forces.^13^, ^19^ To address this challenge a number of recent studies have examined VTCs under flow conditions and optimized their targeting affinity under relevant physiological levels of wall shear stress^20^ (WSS) falling in the broad range^13^, ^21^, ^22^, ^23^ of 1–60 dyne/cm^2^. For example, Ferrante et al^23^ studied microbubble carriers, coupled with ligands to VCAM‐1 and P‐selectin, targeting to atherosclerotic plaque sites under WSS ranges from 1.6–6 dyne/cm^2^. Also, Eniola‐Adefeso et al investigated the effect of carriers' size and ligand coating^21^, ^22^ on their targeting to vascular wall under low WSS ranges between 0.8–2 dyne/cm^2^. In addition to the targeting of VTCs under constant laminar flow conditions, several studies have also focused on VTC targeting under more complex flow conditions, including VTC targeting in low‐shear re‐circulating flows, as such flows are known to occur at atherosclerotic arteries.^22^ Additionally, to improve site specificity, the use of multivalent targeting, using two or more ligands which can provide synergetic adhesion, have also been explored, under these low WSS conditions.^13^, ^23^

Yet, so far these have been limited to healthy or low WSS conditions and have not addressed VTC adhesion under pathological levels of high WSS where WSS can be 100 dyne/cm^2^ and higher.^24^, ^25^ Studies addressing NPs targeting under high WSS region have so far focused on targeting thrombotic arteries^26^ and not the endothelium. However, high WSS and endothelial activation are critical aspects known to occur in several disease conditions including vaso‐constricted arteries or at early stages of arteriogenesis following occlusion of a neighboring artery.^20^, ^24^, ^27^, ^28^ Thus, although not studied before, targeting NPs to the activated EC under these high WSS conditions can be valuable.

In the present work, we explore in vitro the adhesion of VTCs to inflamed endothelium under pathological high shear conditions (>70 dyne/cm^2^) using microfluidic perfusion models. Our results show that 200 nm particles functionalized with activated endothelial ligands, that is, an E‐selectin binding peptide (Esbp) or an anti ICAM‐1 antibody or a combination of both, can selectively target activated endothelial cells under pathological high shear stress conditions. These findings may provide a promising avenue for targeted delivery of therapeutics to sites of diseased and inflamed vasculature, where pathological levels of high WSS are well established.

## 2 MATERIALS AND METHODS

### 2.1 Microfluidic device fabrication

Microfluidic devices were produced from Polydimethylsiloxane (PDMS) using conventional soft‐lithography, as reported previously.^29^ Briefly, a simple channel (22 mm in length and 2 mm in width) was cut in an 80‐μm aluminum adhesive film using a cutter plotter (CE5000, Graphtec, CA). A 2.2 cm long channel was used to assure a uniform, stable WSS at the examined region (see also Computational Fluid Dynamic [CFD] simulation results in Supporting Information and Figure S1). The obtained aluminum strips were placed at the bottom of a petri dish. Next, a PDMS Sylgard 184 (Dow Corning, Midland, MI) mixture containing a 1:10 ratio of crosslinker and resin, respectively was poured into the dish, degassed and kept at room temperature overnight. Afterwards the PDMS was peeled, and inlet and outlet holes were punched. Finally, the PDMS channels were sealed to a glass slide 76 × 50 × 1 mm (Marienfeld, Germany) utilizing oxygen plasma.

### 2.2 Cell culture

Prior to cell seeding, the surface of the microfluidic device was coated using 100 μg/ml human fibronectin (Sigma Aldrich, Israel) in PBS. Human umbilical vein endothelial cells (HUVECs), (Lonza, Israel) were used in all assays. HUVECs were cultured in T75/T150 flasks with ECM media supplemented with FBS, Endothelial Cell Growth Supplement, penicillin/streptomycin solution (ScienCell, Switzerland). HUVECs were trypsinized using trypsin B (Biological Industries, Israel), and 10^6^ cells were seeded in each microfluidic channel. The cells were then incubated at 37°C and 5% CO^2^ with standard media for 2 hr to enable a stable cell adhesion.

Inflammation was induced by activating the HUVECS using 0.1 μg/ml TNF‐α (R&D Systems, MN) in a serum free media, which resulted in E‐selectin and ICAM‐1 over‐expression. The activation was induced under static conditions at 37°C and 5% CO_2_ over different durations (0.5–6 hr). Then the microfluidic channels were washed twice with PBS. Cells were then fixed using 4% paraformaldehyde (Sigma Aldrich, Israel) for 10 min at room temperature, washed three times and stored at 4°C.

### 2.3 Staining HUVEC cells against E‐selectin and ICAM‐1

Cells were stained against E‐selectin by incubating the fixed TNF‐α stimulated cells with 50 μg/ml FITC labeled E‐selectin binding peptide, Esbp^30^ (purchased from GL Biochem, China, >95% purity validated by HPLC and MS),^30^ for 2 hr at room temperature. Also, ICAM‐1 receptors were stained using incubation with 2.5 μg/ml anti ICAM‐1 antibody (ThermoFisher, MA) for 2 hr, and labeled using a secondary antibody Alexa fluor 647 anti‐mouse (ThermoFisher, MA). Cell nuclei were stained using DAPI. The microfluidic channels were washed twice with PBS between staining steps. Confocal images were obtained using a Nikon based confocal microscopy system (Andor, Belfast GB) and recorded by an Andor DS‐Ri2 camera. Images were acquired by an Andor iQ3 software and fluorescent intensity analysis was performed via an ImageJ software. Data was averaged from 3 to 5 images per experiment and three repeat experiments were conducted for each condition.

### 2.4 Particle functionalization

Two hundred nanometers of carboxylated polystyrene particles (ThermoFisher, MA) were modified chemically via EDC/Sulfo‐NHS protocol (Merck, Israel). For Esbp particles, a one‐step process was used, while for anti‐ICAM‐1particles, a NeutrAvidin/Biotin‐Binding method was performed. First, the particles were washed three times with a 2‐(N‐morpholino) ethanesulfonic acid (MES) buffer and incubated with 200 mM EDC and 200 mM Sulfo‐NHS for 30 min at room temperature. At this point, FITC‐Esbp were linked to Esbp particles for 2.5 hr, and NeutrAvidin to aICAM‐1 particles. Then, ethanolamine (Merck, Israel) was added for 30 min to quench the reaction. The particles were washed three times using a suspension in 50 mM Tris, pH 8.0, 0.5% (w/v) casein blocking buffer.

Biotinylated aICAM‐1 was linked to NeutrAvidin coated particles for 2 hr at room temperature. Finally, all particles were washed in PBS contains 1%(w/v) BSA (Millipore, MA) prior to flow adhesion experiments.

The amount of conjugated aICAM‐1 on particle surface was estimated using the supernatant protein content by a Smith assay with bicinchoninic acid kit for protein determination (Sigma Aldrich, Israel). The absorption reads were taken by a CARRY Bio100 spectrophotometer. For particle Esbp quantification, the FITC fluorescence levels were read using a Microplate reader (Varioskan LUX, ThermoFisher, MA). In addition, particle size and zeta potential of coated particles were measured by a DLS Zetasizer Nano instrument (Malvern, GB).

### 2.5 Perfusion system and adhesion experiments

The perfusion system comprises a HUVEC seeded PDMS microfluidic device, a syringe pump (KDS Scientific, MA) connected to a syringe filled with particle solution at a concentration of 3.4 ± 0.5 μg/ml suspended in PBS which was supplemented with 1%(w/v) BSA (Millipore, MA) buffer. Particle concentration was measured via fluorimetry utilizing a calibration curve of the stock solution. The particle solution was perfused through the channel at a controlled constant flow rate ranging between 512 and 3,840 μl/min, to monitor the influence of various levels of WSS (see below). The particle solution was perfused between 15 to 30 min in each experiment. Particle adhesion was assessed via florescent confocal microscopy.

### 2.6 Shear stress and adhesion calculations

Flow‐induced WSS corresponds a tangential force applied on a surface due to viscous fluid flow. In our microfluidic model, we set the WSS using the analytical formula established under laminar flow conditions for a wide channel (*w* ≫ *h*) such that

$$\tau_{w}=\frac{6\mathrm{\mu Q}}{h^{2}w}$$

where *τ*_w_ is the WSS, *μ* the fluid viscosity, *Q* is the flow rate, *h* and *w* are the channel height and width, respectively. Here, we assume a fully‐developed laminar flow for a Newtonian incompressible fluid obeying no‐slip conditions at the wall.

### 2.7 Data analysis and statistics

Confocal time‐lapse images were taken for each flow experiments. Using a custom analysis software (Matlab®), we extract the number of present particles in each frame and the slope representing the average adhesion rate over time (i.e., number of particles per mm^2^ per min). Additionally, the particle adhesion probabilities were also calculated as described in the Suppl. Material. Each flow experiment was repeated between 3–4 times, and 3–6 individual locations in each channel were recorded. Mean data are plotted with corresponding standard deviation (STD) bars and were analyzed as indicated in figure legends. Statistical significance of differences was determined using an unpaired Students *t*‐test. Marks indicate p values of *<.05, **<.01, ***<.001, and n.s. indicates not significant as presented in the figures. All statistical analyses were determined using GraphPad Prism 8® software.

## 3 RESULTS

### 3.1 E‐selectin and ICAM‐1 ligand adhesion to EC

In this work we have focused on VTCs functionalized with two common inflammatory ligands, namely an Esbp and an anti ICAM‐1 antibody. The Esbp is an artificial peptide, first synthesized by Shamay et al.^30^ The Esbp CDITWDQLWDLMK–CONH2 sequence labeled with FITC‐Lys was used in our study to allow its fluorescence detection. The peptide binds E‐selectin with high affinity but not P‐selectin and L‐selectin, members in selectins superfamily.^30^ For I‐CAM1 targeting we used an anti ICAM‐1 monoclonal antibody from mouse origin, which reacts with human ICAM‐1, and has been widely studied for VTCs.^7^, ^31^

As E‐selectin and ICAM‐1 expressions vary as a function of time post stimulation by TNF‐α, we first examined the adhesion of these ligands experimentally in our model at different time points post stimulation. ECs cultured in the microfluidic channels were incubated with TNF‐α and cells were subsequently fixed at various time points following stimulation and then incubated with the examined ligand prior to confocal fluorescent microscopy imaging.

**Figure 2:**
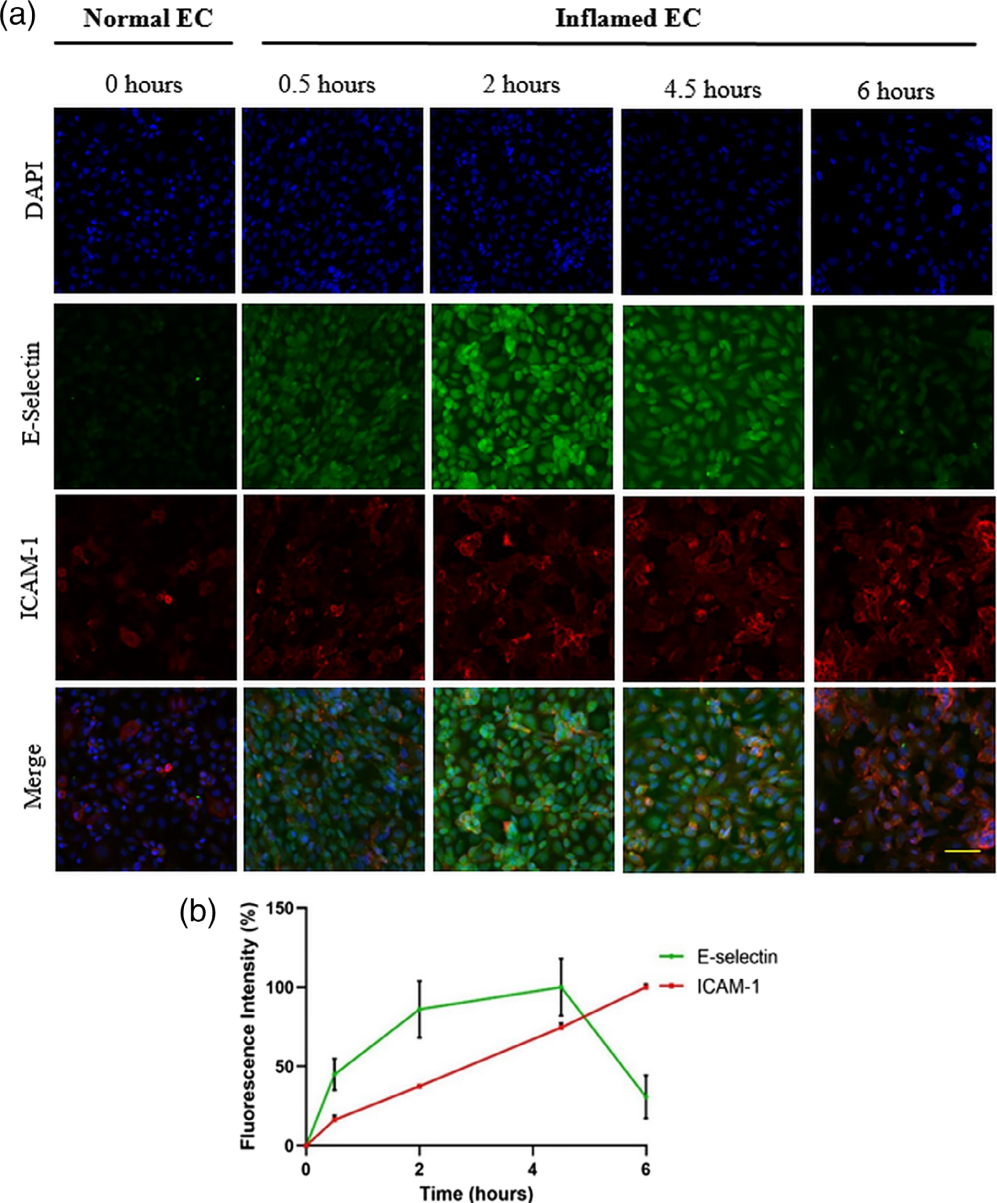
Confocal microscopy images of normal HUVEC cells, and of HUVEC cells inflamed upon stimulation by TNF‐α. (a) The stimulated cells express ICAM‐1 and E‐selectin entities, as revealed by staining with fluorescent‐labeled anti ICAM‐1 antibody (red) and Esbp (green), respectively. Staining by DAPI (blue) served as control. All three are displayed collectively in Merge images. Scale bar: 30 μm. (b) Time course of the normalized fluorescence intensities of cell‐labeled aICAM‐1 (red) and of Esbp (green), as measured in the corresponding images shown in section (a)

Our experiments (see Figure 2) show that the expression of E‐selectin increased as a function time, reaching its peak value at 4 hr of TNF‐α stimulation and then dropped down to almost its basal levels at 6 hr post stimulation. Such results confirm the different kinetics known to exist for these two ligands.^13^ Namely, in the case of ICAM‐1, ICAM‐1 levels increased monotonically as a function of the duration of stimulation by TNF‐α (Figure 2b), reaching their highest value at the longest examined time (6 hr). Within the limitations of the present assay, these results, whereby different ligands exhibit different temporal changes in expression during disease conditions, further suggest that using multivalent targeting via two or more targeting ligands may offer a more robust and effective targeted delivery approach.

### 3.2 Effect of ligand surface density on NPs adhesion under high WSS

**Figure 3:**
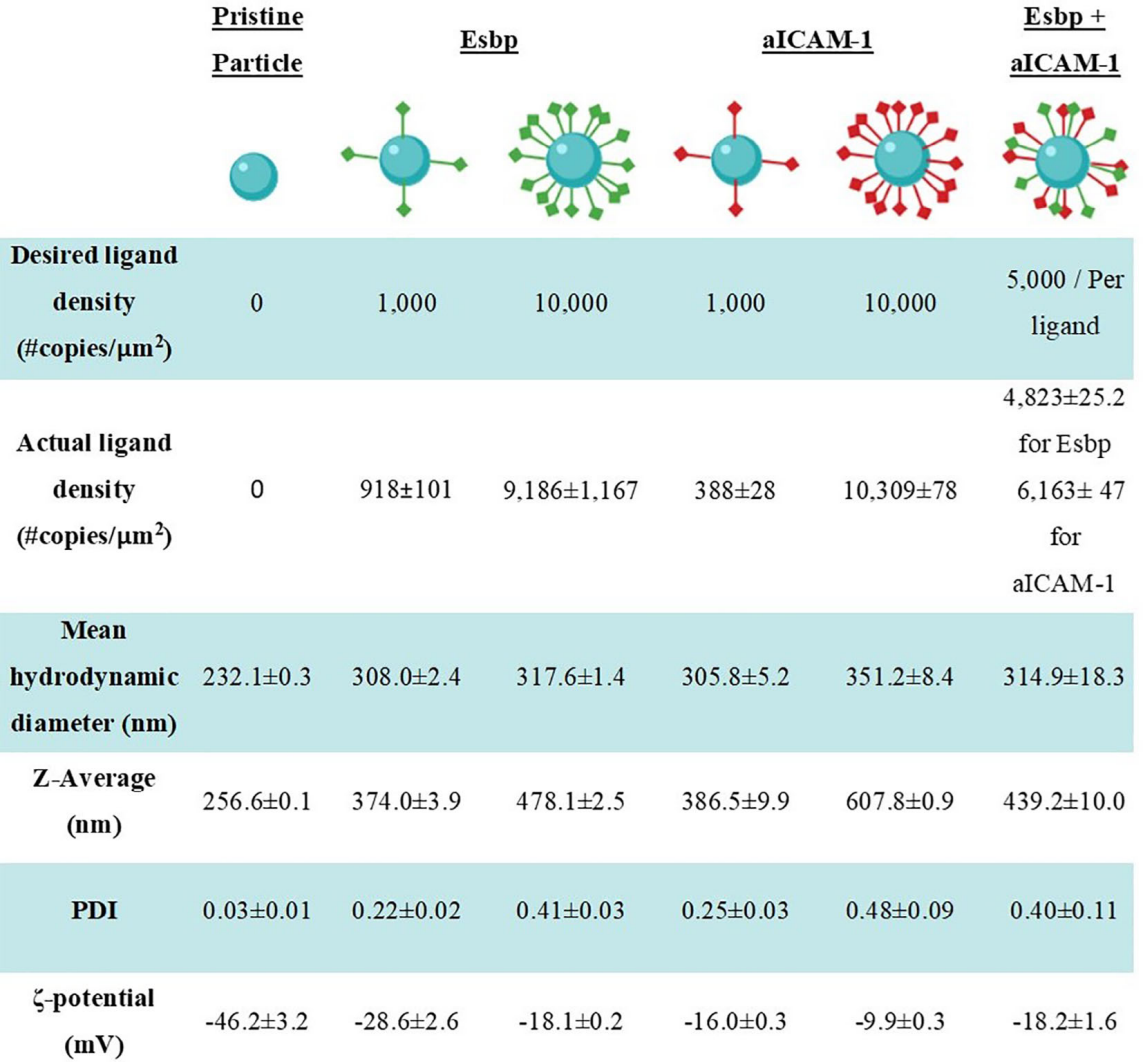
NPs ligand quantification, number mean value and Z‐average of the particles' size, particles' PDI, and the ζ‐potential evaluation

To test the effect of particle surface ligand density on adhesion under high WSS, 200 nm polystyrene NPs were first functionalized at two surface densities: 1,000 (low density) and 10,000 (high density) copies per μm^2^. The ligand density, hydrodynamic diameter and zeta potential of the various studied formulations are summarized in Figure 3. ECs were then cultured in the microfluidic devices and inflamed with TNF‐α over 4 or 6 hr for Esbp and aICAM‐1, respectively. These activation times represent the peak E‐Selectin and ICAM‐1 expression time points^13^ in our experimental system within a <6 hr time frame following the above experiments (Figure 2).

**Figure 4:**
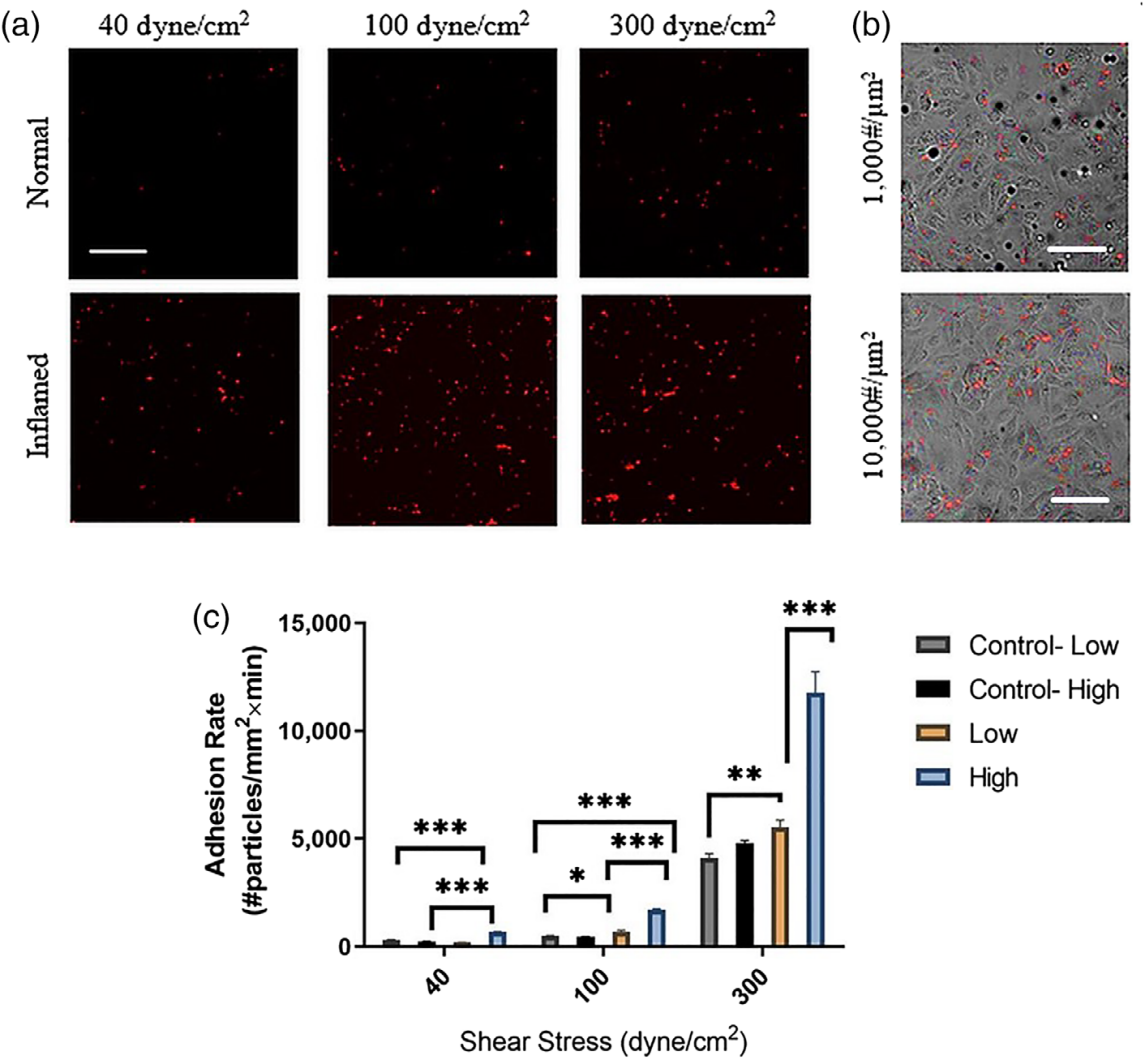
Adhesion of Esbp functionalized nanoparticles (NPs) to activated Endothelial cells (ECs) under high shear stress conditions. (a) Representative fluorescent confocal microscopy images following perfusion experiments over ECs. The images show higher adhesion of Esbp (10,000 copies/μm^2^) functionalized NPs to activated cells, compared to low‐density Esbp NPs (1,000 copies/μm^2^), at for the examined wall shear stresses of: 40, 100, and 300 dyne/cm^2^. Scale bar: 50 μm. (b) Confocal microscopy images comparing the adhesion of Esbp functionalized NPs at two surface densities (1,000 & 10,000 #/μm^2^) to activated ECs (phase images) at a WSS of 300 dyne/cm^2^. High‐density Esbp NPs show greater adhesion to cells. Scale bar: 50 μm. (c) Quantification of the adhesion rate of Esbp‐NPs coated at two densities to activated ECs under flow at different levels of wall shear stress (i.e., 40,100, and 300 dyne/cm^2^). The EC were stimulated by TNF‐α for 4 hr

To begin, high‐density Esbp functionalized NPs were perfused through the microfluidic devices at different WSS: (a) 40 dyne/cm^2^ representing high levels of physiological WSS in arteries, (b) 100 dyne/cm^2^, and (c) 300 dyne/cm^2^ representing pathological levels of WSS. For all the examined levels of WSS, Esbp NPs exhibited selective adhesion to activated cells, while minimal adhesion was noticed in normal unstimulated EC, as shown in Figure 4a. Moreover, we observed that the adhesion rate was shear‐dependent, that is, increasing with higher shear. To test the effect of ligand surface density on adhesion under pathologically high WSS, we performed experiments with the low‐density Esbp NPs and compared them to the high‐density Esbp NPs. The results show a strong trend whereby high density Esbp NPs adhere more than low‐density Esbp NPs, as shown in Figure 4b for representative images at WSS = 300 dyne/cm^2^. Quantitative analysis of the results (Figure 4c) show that at 100 and 300 dyne/cm^2^, the adhesion rate of high ‐density Esbp NPs to activated EC is more than two folds higher compared to low‐density Esbp NPs. Thus, Esbp NPs appear to provide a selective attachment to activate the inflamed ECs and increasing the ligand density has a positive impact on this adhesion.

**Figure 5:**
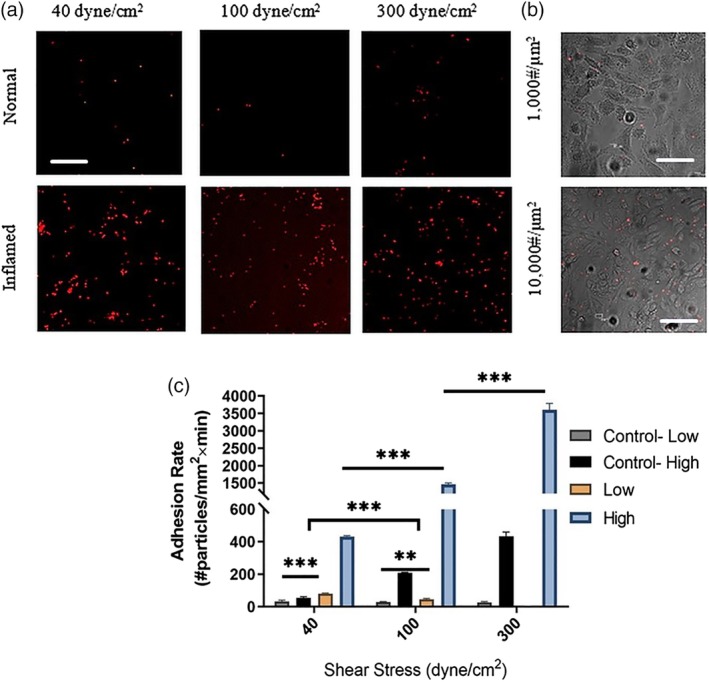
Adhesion of aICAM‐1 functionalized nanoparticles (NPs) to activated Endothelial cells (ECs) under high shear conditions. (a) Representative fluorescent confocal microscopy images following perfusion experiments over ECs. The images show higher adhesion of aICAM‐1 (10,000 copies/μm^2^) NPs to activated cells at the examined wall shear stresses: (i.e., 40, 100, and 300 dyne/cm^2^). Scale bar: 50 μm. (b) Confocal microscopy images comparing the adhesion of aICAM‐1 functionalized NPs at two surface densities (1,000 & 10,000 #/μm^2^) to activated ECs phase image) at a WSS of 300 dyne/cm^2^. High‐density aICAM‐1 particles show greater adhesion to cells. Scale bar: 50 μm. (c) Quantification of the adhesion rate of aICAM‐1‐NPs coated with two densities to activated ECs under flow at different levels of wall shear stress (i.e., 40,100, and 300 dyne/cm^2^). The EC were stimulated by TNF‐α for 6 hr

To test aICAM‐1 NPs, the same perfusion experiments were repeated for conditions of low‐density and high‐density aICAM‐1 NPs; their adhesion under flow to inflamed, (6 hr) TNF‐α stimulated ECs as well as normal ECs was monitored and quantified. For high‐density aICAM‐1 NPs (Figure 5), the selective adhesion to activated ECs compared with unstimulated ECs was consistently observed for all the WSS values examined (i.e., 40, 100, and 300 dyne/cm^2^). In contrast, for low‐density aICAM‐1 NPs, significantly less adhesion was observed under the same WSS conditions, as observed in Figure 5b showing representative confocal microscopy images at WSS of 300 dyne/cm^2^. Quantitative analysis of the adhesion rate of both particles (Figure 5c) underlines a >3‐fold increase in particle binding of high‐density aICAM‐1 NPs, when spanning 100–300 dyne/cm^2^. On the other hand, low‐density aICAM‐1 NPs show a decrease in binding when WSS increases, as well as significantly less overall adhesion. Regarding the high aICAM‐1 results (Figure 5c) the observed behavior of the NPs can be attribute to a more complex interaction between the anti‐body and the ligand, which can also be influenced by flow.

### 3.3 Adhesion of Esbp + aICAM‐1 dual functionalized NPs under high WSS

Given the enhanced adhesion under high WSS of high‐density NPs, for both Esbp and aICAM‐1 NPs, we examined also dual functionalized NPs comprising a combination of Esbp and aICAM‐1 (see functionalized NPs characteristics in Figure 3). Specifically, 5,000 copies of each ligand were immobilized on the same particle to produce the same total molecules as compared under high‐density Esbp and aICAM‐1 NPs. We then perfused the NPs on 0.5‐hr TNF‐α stimulated EC or 4 hr stimulated EC, while monitoring particle adhesion under high WSS. Then we compared them with high‐density Esbp and aICAM‐1 NPs.

**Figure 6:**
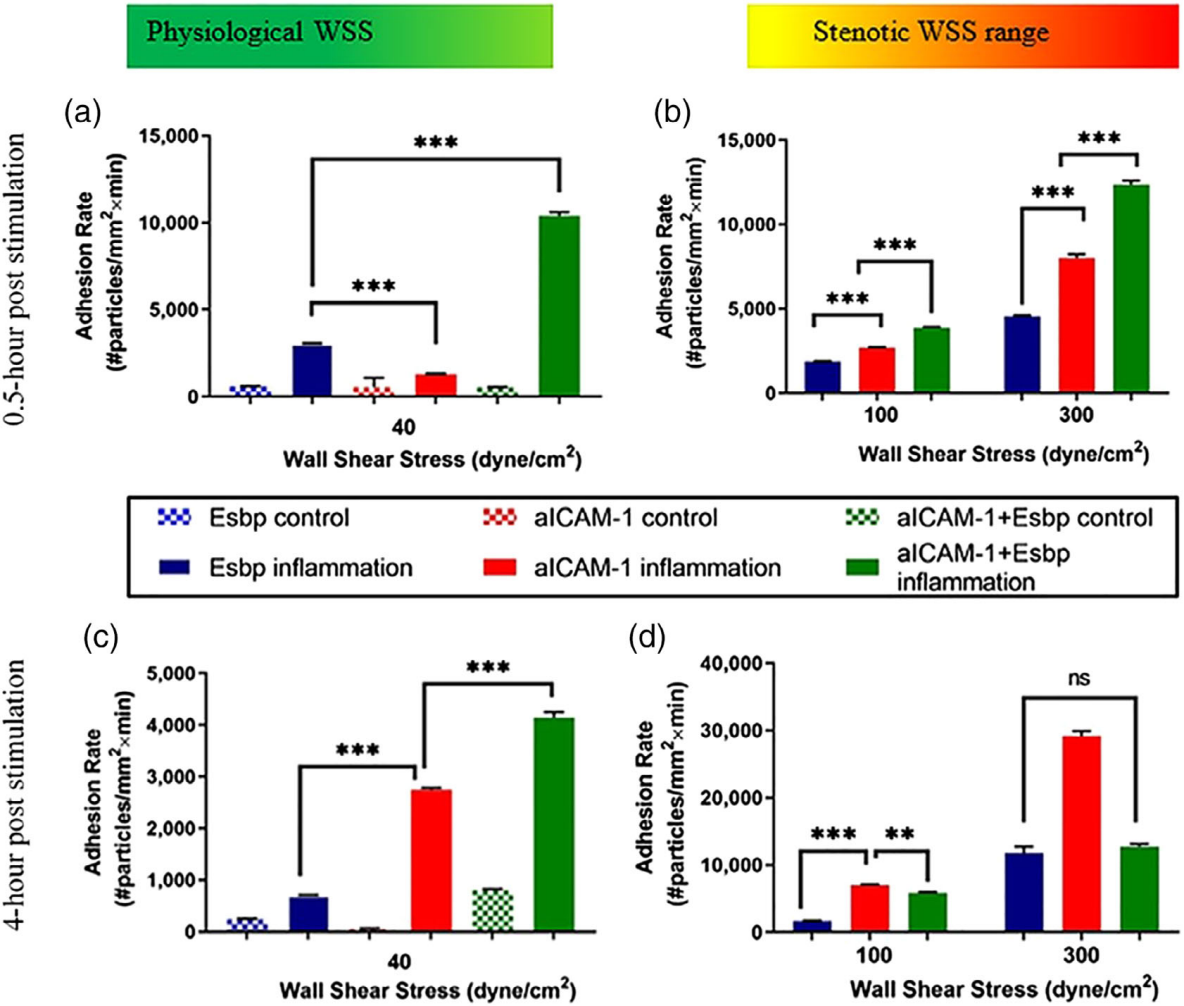
Comparing adhesion rate histograms of Esbp (10,000 #/μm^2^), aICAM‐1(10,000 #/μm^2^), and dual‐functionalized Esbp + aICAM‐1 NPs (total 10,000 #/μm^2^ at a ratio of 1:1) to normal ECs (control) and to activated ECs (TNF‐α stimulated ECs). Half an hour post stimulation by TNF‐α (a) under normal WSS of 40 dyne/cm^2^ and (b) under pathological WSS of 100–300 dyne/cm^2^. Four hours post stimulation by TNF‐α (c) under normal WSS of 40 dyne/cm^2^ and (d) under pathological WSS of 100–300 dyne/cm^2^

As shown in Figure 6a, for 0.5 hr stimulated ECs, under normal physiological levels of WSS (40 dyne/cm^2^) all formulations exhibited enhanced adhesion to activated EC compared to normal EC demonstrating their specific interaction with inflamed EC. Additionally, for all the examined NPs, the adhesion rate reaches a maximum level at the maximum examined (WSS = 300 dyne/cm^2^) as shown in Figure 6b. Thus, the increase in shear stress correlates with an increased flux of particles and thereby results in more particles that adhere. However, as the adhesion interaction between the NPs and the inflamed EC layer under flow are complex in nature, a non‐linear correlation is observed throughout our experiments, even for particles that exhibit high affinity to their target. Additionally, in order to normalize the particle flux, we calculated the adhesion probabilities of the three types of NPs to activated EC under WSS of 40, 100, and 300 dyne/cm^2^ (see Supporting Information and Figures S2 and S3).

Importantly, upon comparing the different particle coatings, particle adhesion of the dual‐targeted NPs produced superior adhesion compared to high‐density Esbp and aICAM‐1 NPs at all WSS values, thus highlighting the synergistic action of Esbp and aICAM‐1 adhesion under the examined condition of 0.5 hr TNF‐α stimulation.

As shown in Figure 6c, when the same NPs were tested after 4 hr of inflammation at physiological levels of WSS = 40 dyne/cm^2^, all of the NPs formulations showed high selectivity to activated ECs. Additionally, although under this physiological level of WSS the adhesion rate of the dual‐targeted NPs was superior to high‐density aICAM‐1 NPs and high‐density Esbp NPs, when NPs were tested under pathological levels of WSS the high‐density aICAM‐1 (Figure 6d) NPs exhibited the highest adhesion rate. This effect was most pronounced at 300 dyne/cm^2^ where a three‐fold increase in the adhesion was observed compared to Esbp NPs and dual targeting NPs. These findings at longer time points post TNF‐ α simulation are in line with previous data^13^, ^20^ obtained at physiological levels of WSS (i.e., in the range of 10–70 dyne/cm^2^), whereby the inflammatory receptors overexpression changes it profile at different time‐points post stimulation.

## 4 DISCUSSION

Our in vitro results confirm that targeting NPs to activated ECs, via E‐selectin and ICAM‐1 receptors, can provide selective adhesion not only at physiological levels of arterial WSS, as described in previous studies, but also and importantly at pathological levels of high WSS, associated with sites of arterial stenosis. The examined functionalized NPs exhibited stable adhesion even at a WSS of 300 dyne/cm^2^, suggesting strong ligand‐receptor linkage. Even at a coating density of 1,000 copies per μm^2^ for both Esbp and aICAM‐1 NPs some enhanced adhesion at high WSS was observed; namely, increasing the coating density to 10,000 copies per μm^2^ resulted in significantly more adhesion. This latter result agrees with the trend that the probability of first ligand‐receptor pairing is higher when there are more ligands on particle surface. Moreover, in this study, the attachment between the particle and the EC layer is stronger when more ligand‐receptor bindings are present. However, an increase in the number of ligands on the particles surface does not always lead to greater adhesion of NPs to cells, and in some cases may have a counterintuitive negative effect as described by Zern et al,^32^ particularly with regards to the trade‐off between NPs targeting and their selectivity, which is highly dependent on the NPs ligand density. Consequently, ligand density is an important parameter that should be adequately studied for optimal targeting of the NPs, including under high WSS conditions associated with several vascular disease scenarios.

As the expression of EC adhesion receptors changes temporally upon exposure to an inflammatory mediator (i.e., TNF‐α in our case), VTCs must be designed correspondingly. Here, specifically we focused on a relatively short TNF‐α stimulation (<6 hr). Nevertheless, within such time interval significant differences exist in the expression of ECs E‐selectin and ICAM‐1 receptors, which affected profoundly the targeting of Esbp and aICAM‐1 NPs under perfusion conditions. The Esbp and aICAM‐1 NPs show different adhesion curves at different inflammation times. This may be the result of the different expression at the examined time points as well as the different dissociation constant of these receptors, which determine how stable the pairing will be for each ligand at a defined shear stress. It is known that E‐selectin is involved in the initial stages of attachment and rolling of leukocytes following the initial adhesion the ICAM‐1 receptors is responsible for the firm bond.^3^ These different bond characteristics are also reflected in the present results. Esbp NPs show superiority in adhesion compared to aICAM‐1 NPs only at the normal physiological shear at 0.5 hr of TNF–α simulation. Interestingly, in spite of its low expression at 0.5 post TNF–a simulation, aICAM‐1 NPs had high levels of adhesion even at high levels of WSS. Following 4 hr of stimulation, aICAM‐1 NPs are superior to Esbp NPs across all the examined WSS including at a physiological level of 40 dyne/cm^2^. The difference between the two particles is more pronounced than at 0.5 post TNF‐α simulation. Thus ICAM‐1 may offer a more firm and stable adhesion compared to E‐selectin and similarly aICAM‐1 NPs may offer stronger adhesion compared to Esbp NPs,^33^ suitable for high WSS conditions.

When dual targeting particles were tested, these particles exhibited significant higher adhesion compared to both Esbp and aICAM‐1 particles after half an hour of inflammation (Figure 4), at all the examined WSSs, and showed a higher adhesion probability under high WSS (100 and 300 dyne/cm^2^, see Figure S2). Thus, suggesting synergetic effects of E‐selectin and ICAM‐1 binding to their ligands. At 4 hr of TNF‐α stimulation, this synergetic effect diminishes probably due to the fact that the inflammatory receptor overexpression profile has changed. Therefore, the ratio of 1:1 does not fit optimally the receptors' expression as ICAM‐1 is significantly more dominant than the E‐selectin.^13^ Additionally, for each ligand a lower surface density, that is, 5,000 copies per μm^2^, on the dual targeting particles exists and as ICAM‐1 interaction becomes dominated the adhesion compared to the higher 10,000 copies per μm^2^ aICAM‐1 is reduced. This phenomena was also well investigated by Eniola‐Adefeso et al^13^ for particle tested under normal WSS. Besides the difference in the ICAM‐1 and E‐selectin receptors', there are also a difference between antibody and peptide functions and stabilities^34^: antibodies are of a larger size than peptides, and exhibit poor tissue penetration. But peptides perform instability, susceptibly to degradation and delivery limitations. In our case, aICAM‐1 and Esbp are of different molecular size (>150 and 2.2 kDa correspondingly) and thus their size and conformation may also affect sterically the ligand‐receptor pairing.

In comparison to previous studies, our work mainly focuses on improving inflamed endothelial cells active‐targeting under high WSS (40–300 dyne/cm^2^) while previous work focused on WSS below 10 dyne/cm^2^. For example, Eniola‐Adefeso et al^13^ showed that dual‐targeted NPs result in an adhesion which is superior to mono‐targeted NPs at WSS of 0.8–2 dyne/cm^2^ and in an inflamed mesentery in vivo model. Our results under high WSS, also show a similar trend where the dual functionalized NPs adhere better compared to the mono‐targeted NPs, at 0.5 hr post TNF‐α stimulation. However, following 4 hr of TNF‐α stimulation, the aICAM‐1 NPs are more effective than the dual formulation highlighting that this synergetic effect depends on the temporal expression of both targeted receptors as well as the WSS. Additionally, our results at high WSS also agree with the previous finding at physiological levels of WSS showing that an increase in the ligand‐density leads to a more firm adhesion.^13^ Indeed, the high density coated NPs adhere better to the inflamed ECs under high WSS, however, as shown in Figure 6, this comes at the cost of a significant increase in the adhesion of the high density coated NPs to non‐inflamed ECs. When looking at the specificity of the different NPs formulation, defined as the ratio between the adhesion of NPs to activated ECs (on target) divided by the adhesion to normal ECs (off‐target), at different levels of WSS (Figure S4) a complex behavior is observed where the coating type, coating density and activation time all play a role. For both Esbp (Figure S4a) and aICAM‐1 NPs (Figure S4b) the higher density NPs correspond to higher specificity at all the examined high WSS. Additionally, the high‐density aICAM‐1 NPs show higher specificity than high‐density Esbp NPs. As shown in Figure S4c,d, increasing the activation time (0.5 vs. 4 hr), leads to significant increase in the selectivity of adhesion, particularly for the aICAM‐1 NPs which at long activation time and high WSS is superior to all the other formulations. Thus, at high WSS optimizing the selective adhesion under flow of functionalized drug carriers is an important task, which needs to be tailored based on the pharmacokinetics and safety profiles of the drug to be delivered. Thus, at high WSS optimizing the selective adhesion under flow of functionalized NPs is an even more challenging task then under physiological conditions.

Although our study provides initial evidence that NPs can be designed and optimized for targeting activated ECs under high pathological levels of WSS, such designed particles will require further investigation under more relevant conditions mimicking specific disease conditions, for example, stenosis, both in vitro and in vivo. Additionally, other ligands may be of interest to study, such as VCAM‐1, and the combination of multiple ligand displays at various ratios on the same NPs requires further investigation.

## 5 CONCLUSIONS

Specific targeting of particles for drug delivery, using inflammatory ligand‐receptor pairing, can be valuable for treatment of a variety of diseases and has thus received considerable attention in the field of cardiovascular nanomedicine. However, most of the work so far focused on targeting under low to normal WSS both in vitro and in vivo.^13^, ^21^, ^22^ Here we show that functionalized particles with anti‐inflammatory ligands can selectively adhere to inflamed endothelial cells under high WSS, which is anticipated in stenosis or in abnormally vaso‐constricted arteries. Further design of such carrier is required in order to tailor such nanomedicine to defined pathological conditions where high WSS occurs.

## CONFLICT OF INTEREST

The authors declare no potential conflict of interest.

## AUTHOR CONTRIBUTIONS

H.Z., M.K., and N.K. conceived the experiments. Y.S. synthesized the Esbp peptide. H.Z. functionalized VTCs with Esbp and aICAM‐1, performed the perfusion experiments with the microfluidic models and analyzed the data. H.Z. and N.K. wrote the manuscript in consultation with Y.S., J.S., and N.L. All authors discussed the results and contributed to the final manuscript.

## Supporting information


**Figure S1:** Illustration of the microfluidic channel and CFD WSS simulation results. (A) The microfluidic channel geometry SolidWorks® model. (B) Channel mesh bottom view, (C) Channel mesh top view. (D) WSS color bar map for microfluidic channel model showing homogenous distribution along the central part of the channel
**Figure S2:** Quantification of the adhesion probability of (A) Esbp (10,000 #/μm^2^), (B) aICAM‐1(10,000 #/μm^2^) and (C) dual‐functionalized Esbp+aICAM‐1 NPs (total 10,000 #/μm^2^ at a ratio of 1:1) to activated ECs at the examined wall shear stresses: (i.e. 40, 100, and 300 dyne/cm^2^). (D) Summary of all adhesion probabilities. The EC were stimulated by TNF‐α for 0.5 hour
**Figure S3:** Quantification of the adhesion probability of (A) Esbp (10,000 #/μm^2^), (B) aICAM‐1(10,000 #/μm^2^) and (C) dual‐functionalized Esbp+aICAM‐1 NPs (total 10,000 #/μm^2^ at a ratio of 1:1) to activated ECs at the examined wall shear stresses: (i.e. 40, 100 and 300 dyne/cm^2^). (D) Summary of all adhesion probabilities. The EC were stimulated by TNF‐α for 4 hours
**Figure S4:** Comparison of the specificity of adhesion of the various NPs formulation as a function of the WSS. Specificity was defined as the ratio between the adhesion of NPs to activated ECs (on target) divided by the adhesion to normal ECs (off‐target). (A) low‐density and high‐ density Esbp NPs following 4hr of TNF‐α stimulation, (B) low‐density and high‐ density aICAM‐1 NPs following 6hr of TNF‐α stimulation; (C) Esbp, aICAM‐1 and dual‐targeted NPs after 0.5 hr of EC activation (D) Esbp, aICAM‐1 and dual‐targeted NPs after 4h of EC activation
