# Supplementary material for: Targeting functionalized nanoparticles to activated endothelial cells under high wall shear stress
Source: Bioeng Transl Med. 2019 Dec 13;5(2):e10151. doi: 10.1002/btm2.10151 (PMC7237145; doi:10.1002/btm2.10151)
Supplement: Supplementary file 1 — Figure S1: Illustration of the microfluidic channel and CFD WSS simulation results. (A) The microfluidic channel geometry SolidWorks® model. (B) Channel mesh bottom view, (C) Channel mesh top view. (D) WSS color bar map for microfluidic channel model showing homogenous distribution along the central part of the channel Figure S2: Quantification of the adhesion probability of (A) Esbp (10,000 #/μm2), (B) aICAM‐1(10,000 #/μm2) and (C) dual‐functionalized Esbp+aICAM‐1 NPs (total 10,000 #/μm2 at a ratio of 1:1) to activated ECs at the examined wall shear stresses: (i.e. 40, 100, and 300 dyne/cm2). (D) Summary of all adhesion probabilities. The EC were stimulated by TNF‐α for 0.5 hour Figure S3: Quantification of the adhesion probability of (A) Esbp (10,000 #/μm2), (B) aICAM‐1(10,000 #/μm2) and (C) dual‐functionalized Esbp+aICAM‐1 NPs (total 10,000 #/μm2 at a ratio of 1:1) to activated ECs at the examined wall shear stresses: (i.e. 40, 100 and 300 dyne/cm2). (D) Summary of all adhesion probabilities. The EC were stimulated by TNF‐α for 4 hours Figure S4: Comparison of the specificity of adhesion of the various NPs formulation as a function of the WSS. Specificity was defined as the ratio between the adhesion of NPs to activated ECs (on target) divided by the adhesion to normal ECs (off‐target). (A) low‐density and high‐ density Esbp NPs following 4hr of TNF‐α stimulation, (B) low‐density and high‐ density aICAM‐1 NPs following 6hr of TNF‐α stimulation; (C) Esbp, aICAM‐1 and dual‐targeted NPs after 0.5 hr of EC activation (D) Esbp, aICAM‐1 and dual‐targeted NPs after 4h of EC activation [file BTM2-5-e10151-s001.docx]

Targeting Functionalized Nanoparticles to Activated Endothelial Cells under High Wall Shear Stress

**Supplementary Material**

*Hila Zukerman, Maria Khoury, Yosi Shammay, Josué Sznitman, Noah Lotan and Netanel Korin*

**Microfluidic Channel Computational Simulation**

Computational Fluid Dynamics (CFD) simulations of the flow field within the microfluidic device model was performed using an Ansys Fluent® software. The flow within the channel was calculated, by solving the laminar Naiver-Stokes equation (Eq.S1) no-slip condition assumed at the wall:

Eq. S1: $\rho\left( \frac{\partial\boldsymbol{u}}{\partial t}+u\cdot\nabla\boldsymbol{u} \right)=-\nabla p+\nabla\cdot(\mu\left( \nabla\boldsymbol{u}+\left( \nabla\boldsymbol{u} \right)^{T} \right))$

Where *ρ* is density, ***u*** is the velocity field, *μ* is the viscosity and *p* is pressure.

The microfluidic channel geometry was built using SolidWorks® software (Figure S1.A) with the dimensions of 22 mm in length, 2 mm in width and 80 μm in height. The diameter of the inlet and outlet is 1 mm. Then the geometry was meshed using Ansys GAMBIT (Figure S1.B, C). The mesh included 1.74 million elements, and the elements used were tetrahedral and a five-layer inflation with 1.2 growth factor was used in the model. We used pressure-based with steady time solver, the simulated fluid was water with the given density (*ρ*) of 998.2 kg/m^3^ and viscosity (*μ*) of 1.0016 (mPa∙s). Boundary conditions were mass flow rate inlet of 1.28 ml/min and zero pressure outlet. Flow rate was similar to the experimental flow rate. The chosen model was laminar flow with no slip condition. The simulation result (Figure S1**)** shows uniform WSS distribution along the microfluidic channel.


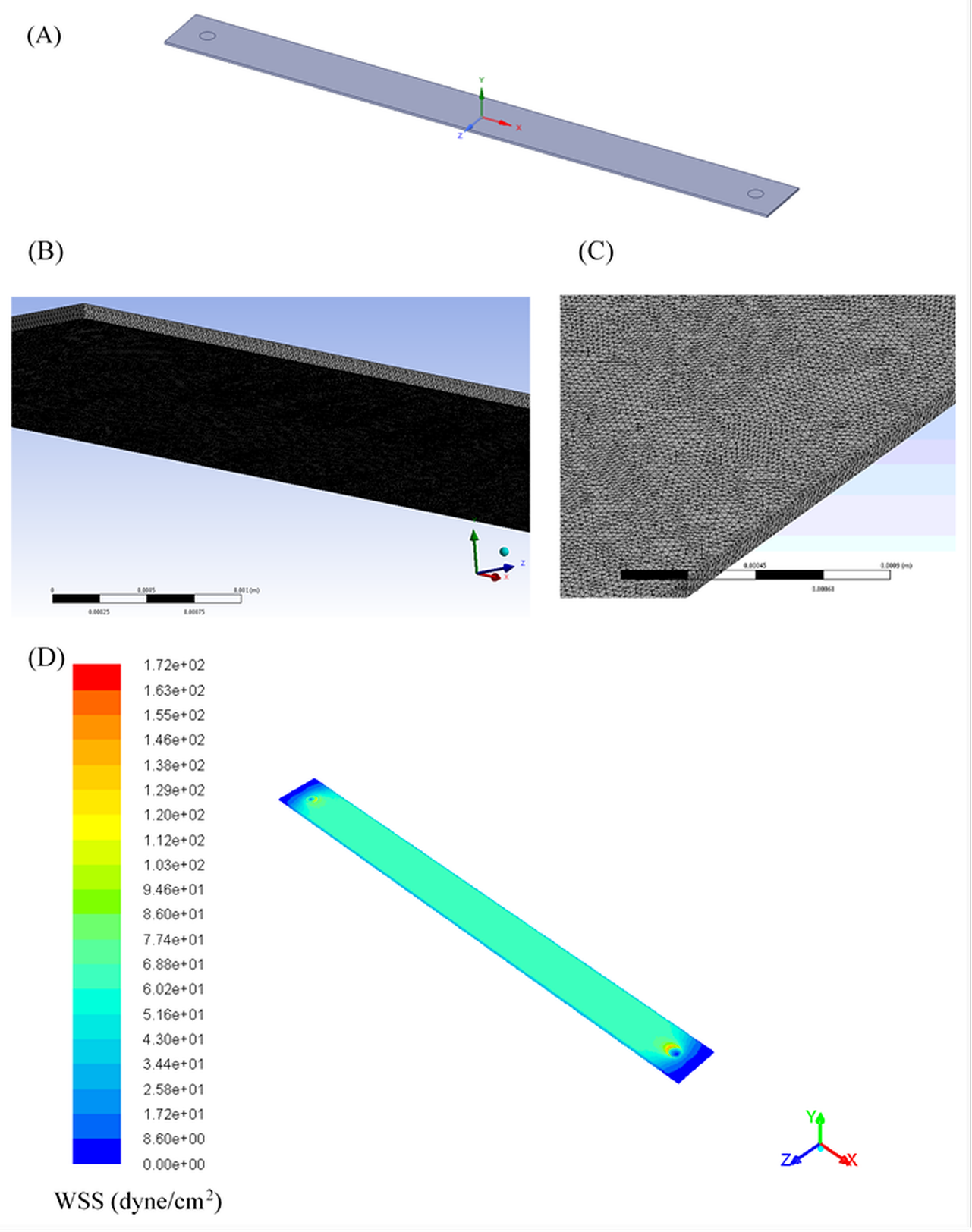


**Figure S1:** Illustration of the microfluidic channel and CFD WSS simulation results. (A) The microfluidic channel geometry SolidWorks® model. (B) Channel mesh bottom view, (C) Channel mesh top view. (D) WSS color bar map for microfluidic channel model showing homogenous distribution along the central part of the channel.

**Nanoparticles (NPs) Adhesion Probability**

The flow experiment data provides NPs adhesion rate under a controlled WSS. However, the different WSS levels were obtained modulating the flow rates, which affect the local NPs flux. Normalization of adhesion rate by the particles’ flux can provide the adhesion probability of NPs, and presents the NPs adhesion specificity under a defined shear level.

The adhesion probability of particles during flow experiments is obtained by Equation S2:

Eq. S2: $Adhesion Probability=\frac{Adhesion Rate}{S\cdot\frac{d_{p}}{2}\cdot C}$

Where $\boldsymbol{S}$ is shear rate, $\boldsymbol{d}_{\boldsymbol{p}}$ is the particle's diameter, and **C** is the particles’ concentration.

The normalized results of NPs adhesion to 0.5-hour-activated EC are shown in Figure S2, and NPs adhesion to 4-hour-activated EC are shown in Figure S3.

**
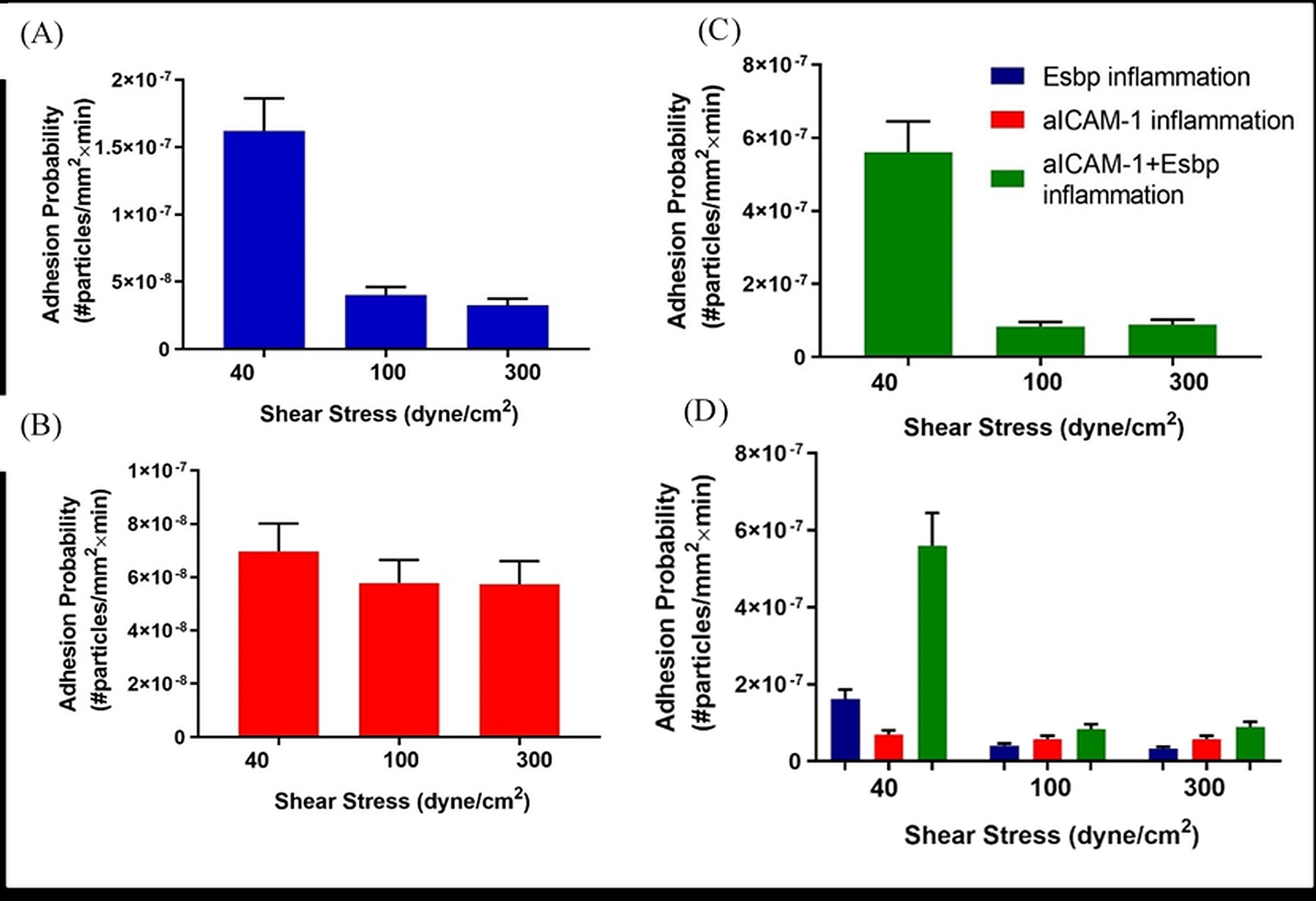
**

**Figure S2:** Quantification of the adhesion probability of (A) Esbp (10,000 #/µm^2^), (B) aICAM-1(10,000 #/µm^2^) and (C) dual-functionalized Esbp+aICAM-1 NPs (total 10,000 #/µm^2^ at a ratio of 1:1) to activated ECs at the examined wall shear stresses: (i.e. 40, 100 and 300 dyne/cm^2­^). (D) Summary of all adhesion probabilities. The EC were stimulated by TNF-α for 0.5 hour.

**
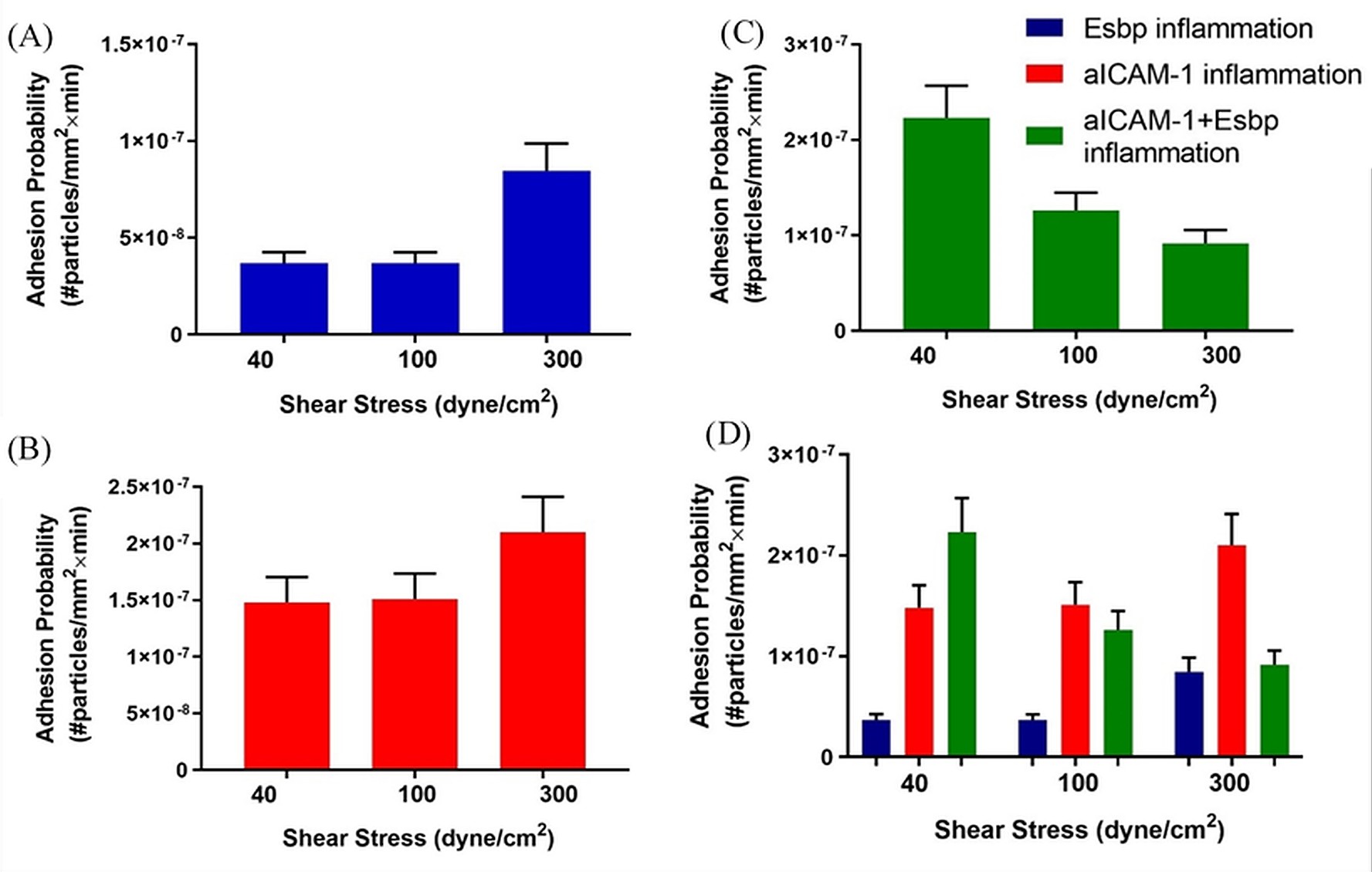
Figure S3:** Quantification of the adhesion probability of (A) Esbp (10,000 #/µm^2^), (B) aICAM-1(10,000 #/µm^2^) and (C) dual-functionalized Esbp+aICAM-1 NPs (total 10,000 #/µm^2^ at a ratio of 1:1) to activated ECs at the examined wall shear stresses: (i.e. 40, 100 and 300 dyne/cm^2­^). (D) Summary of all adhesion probabilities. The EC were stimulated by TNF-α for 4 hours.


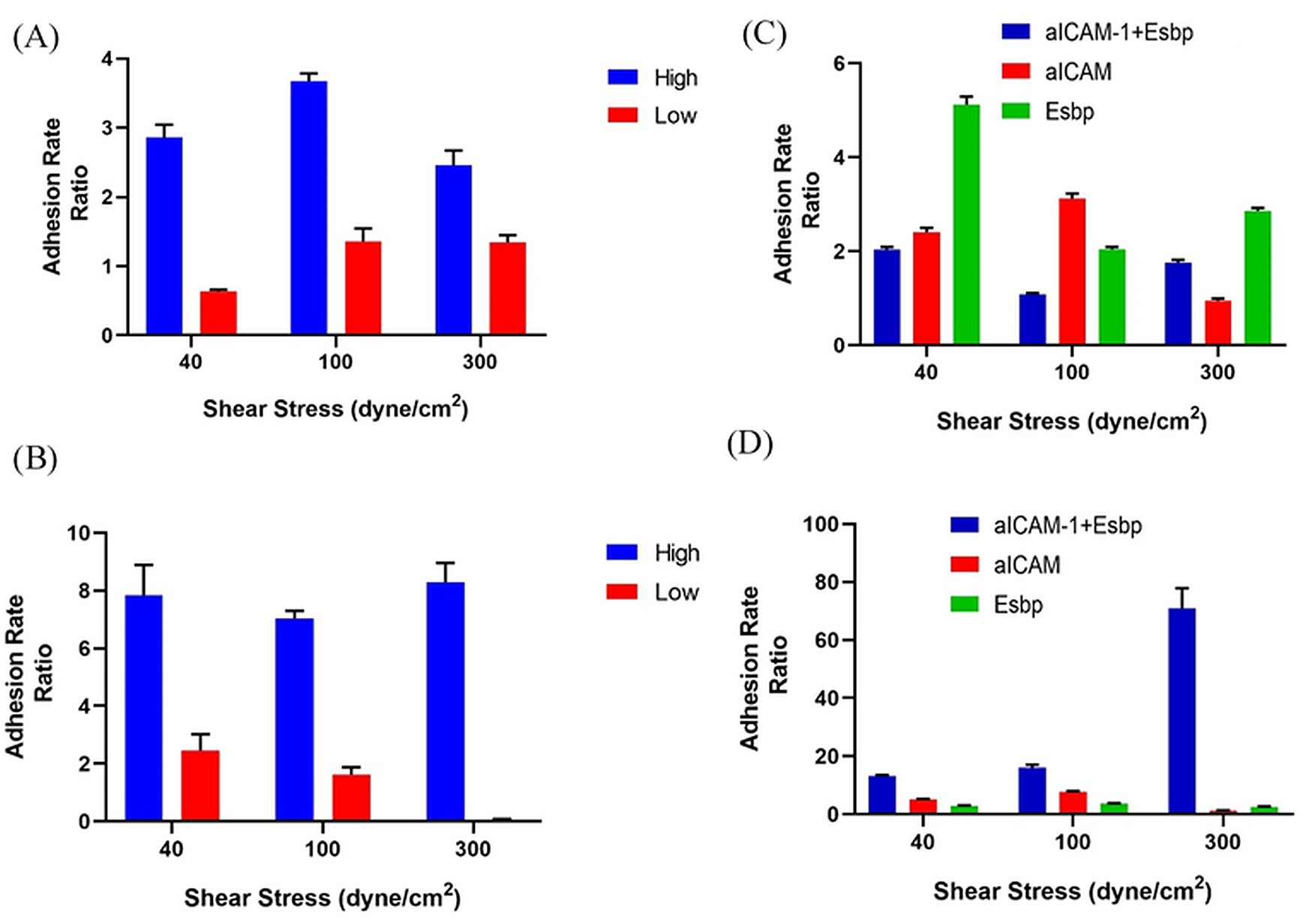


**Figure S4:** Comparison of the specificity of adhesion of the various NPs formulation as a function of the WSS. Specificity was defined as the ratio between the adhesion of NPs to activated ECs (on target) divided by the adhesion to normal ECs (off-target). (A) low-density and high- density Esbp NPs following 4hr of TNF-α stimulation, (B) low-density and high- density aICAM-1 NPs following 6hr of TNF-α stimulation; (C) Esbp, aICAM-1 and dual-targeted NPs after 0.5 hr of EC activation (D) Esbp, aICAM-1 and dual-targeted NPs after 4h of EC activation.
